# Creatine Acts as a Mediator of the Causal Effect of Obesity on Puberty Onset in Girls: Evidence from Mediation Mendelian Randomization Study

**DOI:** 10.3390/metabo14030137

**Published:** 2024-02-25

**Authors:** Chuandi Jin, Guoping Zhao

**Affiliations:** 1 Department of Biostatistics, School of Public Health, Cheeloo College of Medicine, Shandong University, Jinan 250012, China; 2 Microbiome-X, National Institute of Health Data Science of China & Institute for Medical Dataology, Cheeloo College of Medicine, Shandong University, Jinan 250012, China; 3 CAS Key Laboratory of Computational Biology, Bio-Med Big Data Center, Shanghai Institute of Nutrition and Health, University of Chinese Academy of Sciences, Chinese Academy of Sciences, Shanghai 200031, China; 4 Hangzhou Institute for Advanced Study, University of Chinese Academy of Sciences, Hangzhou 310024, China

**Keywords:** Mendelian randomization, mediation analysis, obesity, puberty, Tanner stage, age of menarche, type 2 diabetes, cardiovascular diseases

## Abstract

Epidemiological studies have linked obesity to the onset of puberty, while its causality and the potential metabolite mediators remain unclear. We employed a two-sample Mendelian randomization (MR) design to evaluate the causal effects of obesity on puberty onset and its associated diseases including type 2 diabetes (T2D) and cardiovascular diseases (CVDs). The potential mediators in this pathway were further explored using a two-step MR design. The robustness of our findings was evaluated using sensitivity analyses. Our MR results revealed that childhood obesity/BMI were causally associated with an increased Tanner stage in girls, younger age at menarche, and increased risk of adulthood T2D and CVD. However, neither childhood BMI nor obesity had a causal effect on the Tanner stage in boys. Mediation analysis further indicated that increased creatine served as a mediator for the causal pathway from childhood obesity/BMI to the Tanner stage of girls, while early puberty onset in girls played a mediating role in the pathway linking childhood obesity to increased risk of adulthood T2D and CVD. This study indicated that the risk of early puberty onset in girls and its associated health issues can be potentially reduced by preventing childhood obesity. The involvement of creatine in this process needs to be further validated and explored.

## 1. Introduction

Puberty is a vital milestone in human life, marking the pivotal shift from childhood to the phase of reproductive maturity. Puberty begins with the activation of the hypothalamus–pituitary–gonadal (HPG) axis. The hypothalamus releases pulses of gonadotropin-releasing hormone (GnRH) in pulses, which in turn stimulates the pituitary gland to release luteinizing hormone (LH) and follicle-stimulating hormone (FSH). This cascade of events leads to the development of secondary sexual characteristics and the acquisition of reproductive capacity [1]. Growth hormone (GH) and insulin-like growth factor-1 (IGF-1) also increase markedly during puberty, contributing to the linear growth during this period [1,2]. The secular trend towards earlier puberty worldwide has become a public health concern due to its potential impact on increasing the risk of multiple mental and physical diseases including depression, anxiety, type 2 diabetes (T2D), cardiovascular diseases (CVDs), and reproductive diseases [3]. According to the Tanner staging system, the onset of puberty in girls and boys is marked by the appearance of breast buds (Tanner stage 2, B2) and genital changes (Tanner stage 2, G2), respectively [4]. Meanwhile, the age of menarche has also been utilized as a crucial indicator to determine the onset of puberty in girls [5].

Similarly, childhood obesity is considered to be a major global health problem, and its increasing prevalence has coincided with trends towards earlier pubertal timing [1,6]. Epidemiological studies have linked obesity to the onset of puberty [7], although this association remains controversial. Most of the evidence from case–control, cross-sectional, and longitudinal studies has shown that childhood obesity, higher body fat percentage, and body mass index (BMI) are strongly associated with increased Tanner stage, early menarche age, and risk of precocious puberty in girls [8,9,10]. However, the rate of pubertal development was found to be decelerated in girls with obesity [11]. Inconsistent results have also been reported in boys. While some studies have shown that boys with obesity and higher BMI tend to experience early puberty [12], other studies have found that obesity is not correlated with puberty onset in boys [13,14]. In addition, some studies linked obesity to delayed puberty in boys [15,16]. Furthermore, emerging studies have highlighted the shared potential health risks associated with both childhood obesity and early puberty timing, particularly in relation to an increased risk of T2D and CVD [17,18,19,20].

The potential mechanisms linking childhood obesity with earlier pubertal onset in girls are not fully understood. However, it has been observed that both childhood obesity and early puberty are associated with disturbances in energy homeostasis, hormonal balance, and common metabolic risk factors including insulin resistance, hyperglycemia, and hyperinsulinemia [1,21]. Disruptions in energy sensors, such as inhibition of AMP-activated protein kinase (AMPK) and activation of the mammalian target of rapamycin (mTOR) in the hypothalamus, have been associated with both obesity and early onset of puberty. Leptin and adiponectin, hormones secreted by adipocytes, play pivotal roles in regulating energy balance and have the potential to establish connections between obesity and early puberty onset [22]. Leptin suppresses hunger and enhances energy expenditure, promoting the expression of kisspeptin in the hypothalamus [22,23]. This, in turn, triggers the release of GnRH, potentially leading to earlier puberty onset. In contrast, adiponectin regulates insulin sensitivity and inhibits the GnRH neurons through AMPK pathways [22,24]. Increased leptin and decreased adiponectin have been found in both obese children and children with early onset of puberty [23,24,25]. Furthermore, studies have shown that obesity-related insulin resistance and compensatory hyperinsulinemia contribute to the HPG axis activation and increased leptin and LH levels [23,26]. Conversely, reduced insulin levels and insulin receptors’ deficiency have been associated with suppressed GnRH levels and delayed puberty development [23,27]. In addition, hyperinsulinemia may potentially affect the timing of puberty by reducing sex hormone-binding globulin concentrations and increasing the bioavailability of sex steroids in overweight children [28]. It can also stimulate LH-induced and adrenal androgen production, leading to elevated androgen levels, which have been shown to have a potential effect on earlier puberty in girls by promoting GnRH secretion [1,29]. Furthermore, a high-fat diet as a risk factor for obesity has been linked to early puberty, possibly induced by elevated serum levels of estradiol and leptin, and the activation of GnRH via hypothalamic microglial cells and phoenixin action [30]. Moreover, dysregulated metabolites, such as glucose, lipids, and amino acids, have been implicated in both obesity and puberty development [31,32,33,34,35], while there is still limited understanding of the potential role of metabolites in mediating the link between obesity and puberty.

Most of the empirical data considering the relationship between obesity and pubertal development stem from observational studies and rodent experiments, which carry the potential risk of spurious associations and reverse causation due to confounding, and may not reflect the causal relationship between obesity and puberty in humans. To address these issues, Mendelian randomization (MR) analysis is an instrumental-variable method that leverages genetic variants to obtain causal estimates [36]. The core principle of MR is the random allocation of genetic variants during meiosis, which mimics a randomized controlled trial. In recent years, MR has garnered considerable attention due to its ability to provide more robust evidence of causality than the conventional observational studies [37]. Two-sample MR offers the advantage of utilizing summary-level data from multiple, independent sources, thereby increasing the sample size and statistical power of the causal estimate while minimizing the risk of bias associated with overfitting or selection bias. In addition, two-stage MR (also known as network MR) analysis has emerged as a popular method for conducting mediation analyses to investigate potential mediation effects of an intermediate characteristic between exposure and outcome.

To test whether childhood obesity is a causal factor affecting the pubertal onset and associated diseases, we employed a two-sample MR method to evaluate the causal effect of both childhood BMI and obesity on the age at menarche, Tanner stage of girls and boys, and risk of adulthood T2D and CVD. Furthermore, we utilized a two-step MR design to examine whether early puberty onset serves as a mediator in the association between childhood obesity and increased risk of T2D and CVD while also exploring the mediation effect of metabolites between obesity and pubertal development.

## 2. Materials and Methods

### 2.1. Data Sources

**Figure 1:**
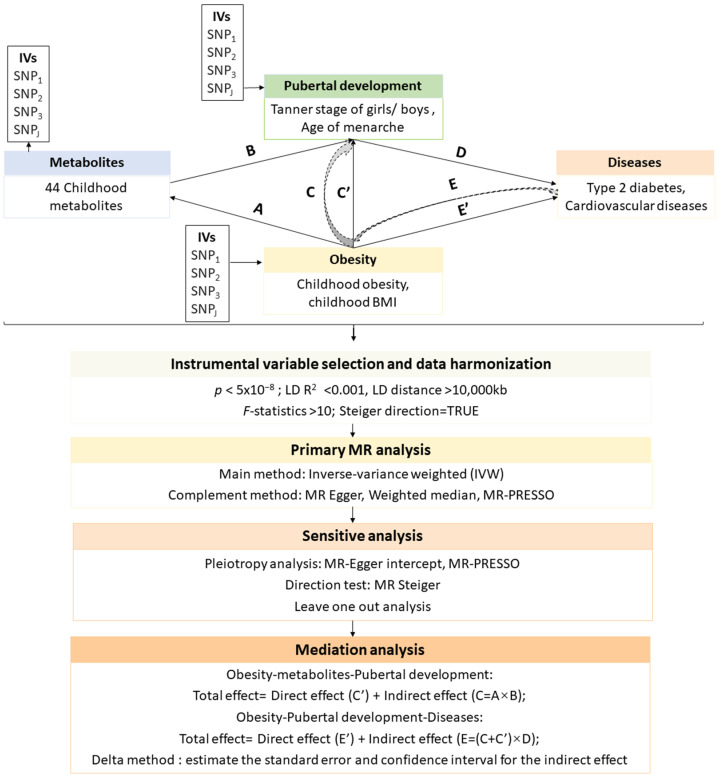
Study design. The causal effects of childhood obesity/BMI on the age at menarche, Tanner stage of girls and boys, and the risk of adulthood type 2 diabetes and cardiovascular diseases were evaluated using a two-sample Mendelian randomization method. The robustness of the Mendelian randomization results were further assessed through a series of sensitivity analyses. Additionally, a two-step Mendelian randomization design was employed to conduct mediation analysis, exploring the mediation effect of pubertal development between obesity and the risk of type 2 diabetes and cardiovascular diseases, as well as investigating the mediation effect of metabolites between childhood obesity and pubertal development.

Figure 1 displays the study design. Summary statistics data were sourced from genome-wide association studies (GWAS) in European ancestry. Data of childhood BMI were obtained from the Early Growth Genetics Consortium (ECG, http://egg-consortium.org/, (accessed on 10 October 2023)), and included 39,620 individuals. Data of childhood obesity were also obtained from the EGG dataset, comprising 5530 individuals with obesity and 8318 controls [38]. The pubertal development in this study was evaluated based on the Tanner stage and age of menarche. The data on Tanner stage, quantitatively assessed on a scale of 1 to 5, for girls (N = 6147, breast stage) and boys (N = 3769, genital stage), were obtained from the EGG database [39]. The age of menarche data (N = 243,944) was sourced from the GWAS summary data provided by the MRC-IE (http://www.ewascatalog.org/ (accessed on 10 October 2023)). Data on 44 childhood urine metabolite levels were obtained from the Human Early Life Exposome (HELIX, https://helixomics.isglobal.org/ (accessed on 10 October 2023)) [40]. Data for T2D (38,841 cases and 451,248 controls) and CVD (146,524 cases and 312,800 controls) were obtained from the GWAS Catalog (https://www.ebi.ac.uk/gwas/ (accessed on 15 February 2024)) with the accession IDs GCST90018926 and GCST90029019, respectively. Additional details are shown in Table 1.

### 2.2. Instrumental Variable Selection and Data Harmonization

For the MR analysis, instrumental variables (IVs) of exposure were selected based on the following procedure (Figure 1). Firstly, we filtered single-nucleotide polymorphisms (SNPs) that were significantly linked to exposure (*p* < 5 × 10^−8^). Secondly, SNPs were pruned to ensure their independence (clumping distance = 10,000 kb, r^2^ = 0.001). Thirdly, the *F*-statistic of SNPs was calculated [41]. SNPs without weak IV bias were retained to serve as IVs (*F*-statistics > 10). Data harmonization was further conducted based on the outcome data and selected IVs of each exposure. Finally, we performed the MR Steiger analysis to test each identified IV. Only data showing the true MR Steiger direction were retained for subsequent MR analysis.

### 2.3. Primary MR Analysis

Two-sample MR analysis was used to estimate the causal effects of obesity (childhood obesity and BMI) on pubertal development (Tanner stage of girls and boys, age at menarche), childhood metabolites, and the risk of T2D and CVD (Figure 1). The inverse-variance weighted (IVW) method served as the main MR method. We firstly obtained the Wald ratio estimates of each IV. Subsequently, these Wald ratios were meta-analyzed using random effects to obtain the combined causal estimate. The IVW method can obtain reliable estimates when all IVs used in the MR analysis are valid. We retained MR associations with a false discovery rate (FDR) < 0.05 for further investigation. Additionally, several MR methods including the MR-PRESSO (Mendelian Randomization Pleiotropy RESidual Sum and Outlier), Weighted median, and MR-Egger approach were also conducted as a complement to the IVW. These approaches provide alternative strategies for obtaining unbiased causal estimates by allowing for pleiotropy to varying degrees.

### 2.4. Mediation Analysis

We used a two-step MR design to explore the mediation effect of puberty development in the causal pathway from obesity to the risk of T2D and CVD as well as the mediation effect of metabolites in the causal pathway from obesity to pubertal development (Figure 1). This design allowed us to decompose the total effect of obesity on pubertal development into the direct effect of obesity on pubertal development (C′ in Figure 1) and the indirect effect (also known as mediation effect) mediated by obesity through the metabolites (A × B in Figure 1). Meanwhile, the total effect of obesity on the risk of diseases (T2D and CVD) was divided into the direct effect of obesity on the risk of T2D and CVD (E′ in Figure 1) and the indirect effect mediated by obesity through pubertal development, denoted by (C + C′) × D (Figure 1). The percentage of mediation effect was calculated. With the delta method, 95% confidence intervals (CI) and standard error (SE) for the mediation effect were calculated.

### 2.5. Sensitive Analysis

To address the issue of pleiotropy, the MR-Egger intercept method as well as the MR-PRESSO method were conducted in this study. The MR-Egger method utilizes the intercept to represent the average pleiotropic effect across IVs, while the slope coefficient represents the causal estimates after adjusting for pleiotropy. The MR-PRESSO method starts by testing for the presence of horizontal pleiotropic outliers using a global test statistic based on the residuals obtained from regressing the IVs on the outcome. If significant outliers are detected, the method then uses a correction procedure to remove these outliers and obtain a corrected causal effect estimate. To assess the causal direction of MR analysis, the MR Steiger method was employed. The term “TRUE” in MR Steiger results indicates the causal relationship that aligns with the expected direction. We conducted leave-one-out analysis to assess whether any individual IVs had a disproportionate impact on the obtained causal estimates. It involved a stepwise exclusion of one SNP at a time, with subsequent comparison of the estimates derived from each exclusion to those obtained using all instrumental variables (IVs). Significant changes in the outcome upon excluding a specific SNP suggest its potential influence on the overall findings. If the exclusion of a specific SNP leads to significant changes in the outcome, this suggests that the association may be sensitive to that SNP, highlighting its potential influence on the overall results.

### 2.6. Statistical Analysis

All statistical analyses were conducted using R (version 4.2.2). The MR-PRESSO method was conducted using the MRPRESSO (version 1.0) R package,. IVs’ selection, data harmonization, primary MR analysis, and sensitivity analyses were performed with the TwoSampleMR (version 0.5.8) R package. Mediation analysis was conducted using the RMediation (version 1.2.2) R package, while forest plots were obtained with the forestplot (version 3.1.1) R package. 

## 3. Results

### 3.1. Childhood Obesity Causally Promoted Pubertal Development in Girls

**Figure 2:**
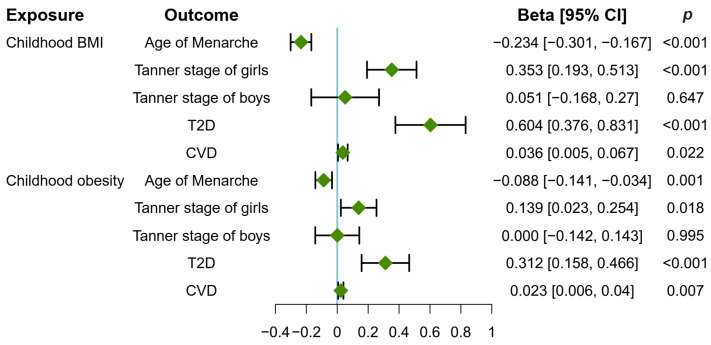
Causal effects of childhood obesity on pubertal development and associated diseases. The forest plot displays the causal estimate (Beta) calculated using the inverse-variance weighted method with random effects, indicated by green diamonds, while the error bars represent the 95% confidence interval (CI). T2D, type 2 diabetes; CVD, cardiovascular disease.

As shown in Figure 2, both childhood obesity and childhood BMI exert a positive effect on pubertal development in girls. Each standard deviation (SD) of higher childhood BMI (β: 0.353, 95% CI: 0.193, 0.513, *p* < 0.001, IVW) and increased childhood obesity (β: 0.139, 95% CI: 0.023, 0.254, *p* = 0.018, IVW) were correlated with an increase in the Tanner stage of girls. Furthermore, childhood BMI (β: −0.234, 95% CI: −0.301, −0.167, *p* < 0.001, IVW) and childhood obesity (β: −0.088, 95% CI: −0.141, −0.034, *p* = 0.001, IVW) were found to contribute to the younger age at menarche. However, neither childhood BMI nor childhood obesity had a causal effect on Tanner stage in boys (*p* > 0.05).

### 3.2. Childhood Obesity Causally Increased T2D and CVD Risk

Childhood obesity and childhood BMI were found to increase the risk of adulthood T2D and CVD (Figure 2). The SDs of higher childhood BMI (β: 0.604, 95% CI: 0.376, 0.831, *p* < 0.001, IVW) and increased childhood obesity (β: 0.312, 95% CI: 0.158, 0.466, *p* < 0.001, IVW) correlated with the increased risk of T2D. Similarly, childhood BMI (β: 0.036, 95% CI: 0.005, 0.067, *p* = 0.022, IVW) and childhood obesity (β: 0.023, 95% CI: 0.006, 0.04, *p* = 0.007, IVW) contributed to the CVD risk.

### 3.3. Early Puberty in Girls Causally Increased T2D and CVD Risk

**Figure 3:**
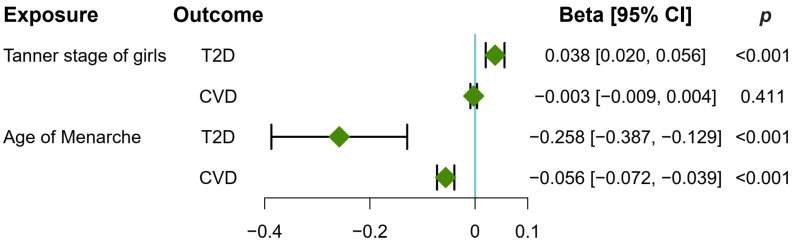
Causal effects of pubertal development on T2D and CVD risk. The forest plot displays the causal estimate (Beta) calculated using the inverse-variance weighted method with random effects, indicated by green diamonds, while the error bars represent the 95% confidence interval (CI). T2D, type 2 diabetes; CVD, cardiovascular disease.

Figure 3 shows that early puberty in girls causally increased the risk of T2D and CVD. Specifically, the increased Tanner stage of girls was causally associated with an increased risk of T2D (β: 0.038, 95% CI: 0.020, 0.056, *p* < 0.001, IVW), while delayed age at menarche reduced the risk of both T2D (β: −0.258, 95% CI: −0.387, −0.129, *p* < 0.001, IVW) and CVD (β: −0.056, 95% CI: −0.072, −0.039, *p* < 0.001, IVW).

### 3.4. Causal Effect of Childhood Obesity on Metabolites

**Figure 4:**
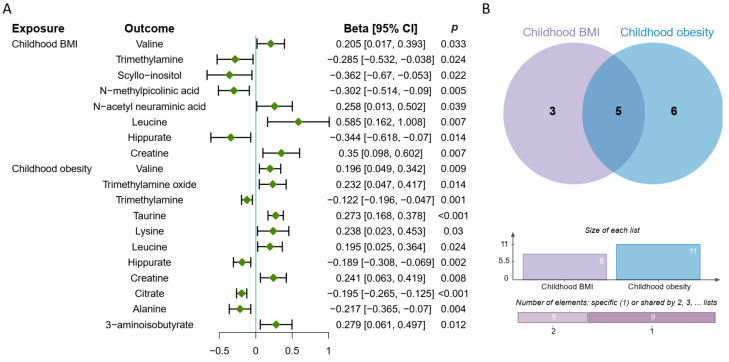
Causal effects of obesity on metabolites in girls and boys. (**A**) The forest plot displays the causal estimate (Beta) calculated using the inverse-variance weighted method with random effects, indicated by green diamonds, while the error bars represent the 95% confidence interval (CI). (**B**) Five common metabolites that were causally affected by both childhood BMI and obesity. Purple and blue represent the metabolites causally influenced by childhood BMI and obesity, respectively.

Figure 4A shows that childhood BMI and obesity were causally associated with 8 and 11 urinary metabolites, respectively. Five common metabolites were causally affected by both childhood BMI and obesity (Figure 4A,B). Among these, childhood BMI and obesity had a promoting effect on the levels of three metabolites including valine (β: 0.205, 95% CI: 0.017, 0.393, *p* = 0.033; β: 0.196, 95% CI: 0.049, 0.342, *p* = 0.009, IVW), leucine (β: 0.585, 95% CI: 0.162, 1.008, *p* = 0.007; β: 0.195, 95% CI: 0.025, 0.364, *p* = 0.024, IVW), and creatine (β: 0.35, 95% CI: 0.098, 0.602, *p* = 0.007; β: 0.241, 95% CI: 0.603, 0.419, *p* = 0.008, IVW) in urine, whereas it contributed to the reduction in trimethylamine levels (β: −0.285, 95% CI: −0.532, −0.038, *p* = 0.024; β: −0.122, 95% CI: −0.196, −0.047, *p* = 0.001, IVW) and hippurate (β: −0.344, 95% CI: −0.618, −0.07, *p* = 0.014; β: −0.189, 95% CI: −0.308, −0.069, *p* = 0.002, IVW). In addition, higher BMI had a negative effect on the levels of scyllo-inositol (β: −0.362, 95% CI: −0.67, −0.053, *p* = 0.022, IVW) and N-methylpicolinic acid (β: −0.302, 95% CI: −0.514, −0.09, *p* = 0.005, IVW), while a positive effect was observed on the levels of N-acetylneuraminic acid (β: 0.258, 95% CI: 0.13, 0.502, *p* = 0.039, IVW). Childhood obesity was associated with increased levels of trimethylamine oxide (β: 0.232, 95% CI: 0.047, 0.417, *p* = 0.014, IVW), taurine (β: 0.273, 95% CI: 0.168, 0.378, *p* < 0.001, IVW), lysine (β: 0.238, 95% CI: 0.023, 0.453, *p* = 0.03, IVW), and 3-aminoisobutyrate (β: 0.279, 95% CI: 0.061, 0.497, *p* = 0.012, IVW), while showing a reducing effect on the levels of citrate (β: −0.195, 95% CI: −0.265, −0.125, *p* < 0.001, IVW) and alanine (β: −0.217, 95% CI: −0.365, −0.07, *p* = 0.014, IVW).

### 3.5. Causal Effect of Metabolites on Pubertal Development

**Figure 5:**
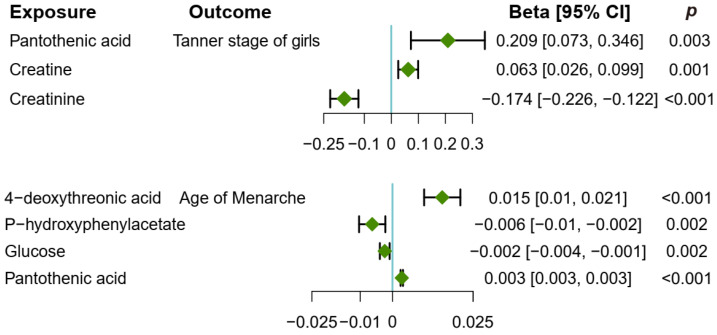
Causal effects of metabolites on pubertal development in girls. The forest plot displays the causal estimate (Beta) calculated using the inverse-variance weighted method with random effects, indicated by green diamonds, while the error bars represent the 95% confidence interval (CI).

Figure 5 shows the significant causal effects of metabolites on pubertal development. Specifically, creatine (β: 0.063, 95% CI: 0.026, 0.099, *p* = 0.001, IVW) was found to be causally associated with an increased Tanner stage of girls, while creatinine, a product of creatine metabolism, was found to have a causal effect on a decreased Tanner stage of girls (β: −0.174, 95% CI: −0.226, −0.122, *p* < 0.001, IVW). Pantothenic acid (β: 0.209, 95% CI: 0.073, 0.346, *p* = 0.003, IVW) also contributed to the increased Tanner stage of girls. Metabolites had weak causal effects on the age of menarche. Two metabolites including 4-deoxythreonic acid (β: 0.015, 95% CI: 0.01, 0.021, *p* < 0.001, IVW) and pantothenic acid (β: 0.003, 95% CI: 0.003, 0.003, *p* < 0.001, Wald) had a delaying effect on the menarche age, whereas, both p-hydroxyphenylacetate (β: −0.006, 95% CI: −0.01, −0.002, *p* < 0.001, IVW) and glucose (β: −0.002, 95% CI: −0.004, −0.001, *p* < 0.001, IVW) were associated with a younger age at menarche.

### 3.6. Puberty in Girls Mediates the Causal Pathway from Childhood Obesity to T2D and CVD Risk

Puberty in girls played a mediating role in the increased risk of T2D and CVD induced by childhood BMI/obesity (Table S1). Specifically, childhood BMI and childhood obesity were causally associated with increased risk of T2D and CVD (Figure 4A). The Tanner stage of girls mediated the causal pathway from childhood BMI (β: 0.013, 95% CI: 0.005, 0.023; *p* = 0.003) and childhood obesity (β: 0.005, 95% CI: 0.001, 0.011; *p* = 0.044) to increased risk of T2D, with the mediation effect accounting for 2.22% and 1.69% of the total effect, respectively. Similarly, age of menarche was found to mediate the causal pathway from childhood BMI (β: 0.060, 95% CI: 0.028, 0.098; *p* = 0.001) and childhood obesity (β: 0.022, 95% CI: 0.007, 0.043; *p* = 0.015) to increased risk of T2D, with the mediation effect accounting for 10.0% and 7.26% of the total effect, respectively. Moreover, age of menarche mediated the causal pathway from childhood BMI (β: 0.013, 95% CI: 0.008, 0.019; *p* < 0.001) and childhood obesity (β: 0.004, 95% CI: 0.002, 0.009; *p* = 0.004) to increased risk of CVD, with the mediation effect accounting for 36.1% and 21.1% of the total effect, respectively.

### 3.7. Creatine Mediates the Causal Pathway from Childhood Obesity to Pubertal Development in Girls

**Figure 6:**
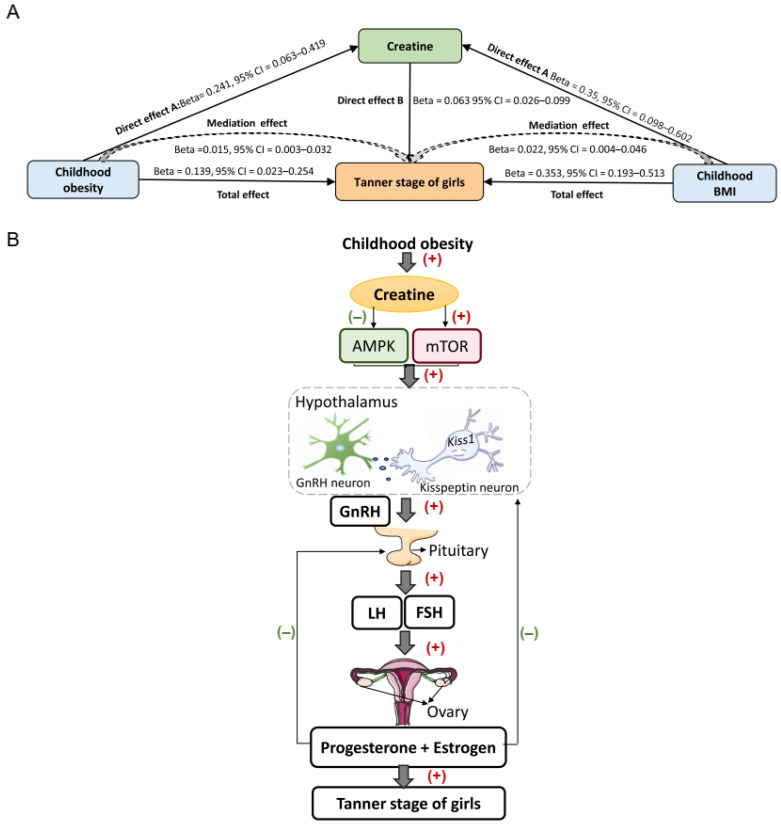
Mediating role and hypothesized mechanisms of creatine in the causal pathway from obesity to puberty onset in girls. (**A**) Creatine partially mediated the causal effect of childhood obesity/BMI on increased Tanner stage of girls. (**B**) The proposed mechanism explaining the mediating role of creatine in the relationship between obesity and early puberty onset in girls is as follows: Elevated levels of creatine induced by obesity may suppress AMPK activity while activating the mTOR pathway. This, in turn, could lead to an upregulation of *kiss1* gene expression, resulting in increased secretion of GnRH, LH, and FSH. These processes promote pubertal development, as evidenced by an advancement in the Tanner stage of girls. The symbols “(+)” and “(–)” are used to denote activation and inhibition, respectively. AMPK, AMP-activated protein kinase; mTOR, mammalian target of rapamycin; GnRH, gonadotropin-releasing hormone; LH, luteinizing hormone; FSH, follicle-stimulating hormone.

Creatine was found to serve as a mediator between childhood BMI/obesity and the Tanner stage of girls, with elevated levels of creatine being causally associated with an increase in Tanner stages in girls (Figure 6A and Table S1). Specifically, significant positive associations were found between childhood BMI/obesity and higher creatine levels (Figure 4A). Moreover, elevated creatine levels mediated the causal pathway from childhood BMI/obesity to an increase in the Tanner stage in girls (Figure 2). The mediation effect of creatine accounted for 6.21% and 10.87% of the total effect of childhood BMI and obesity on increased Tanner stage of girls, respectively. The mediation effects of creatine were 0.022 (95% CI: 0.004, 0.046; *p* = 0.040) for childhood BMI and 0.015 (95% CI: 0.003, 0.032; *p* = 0.043) for childhood obesity.

### 3.8. Sensitivity Analysis

All the IVs and harmonized data are displayed in Table S2. The MR-Egger intercept analysis detected no horizontal pleiotropy in this study (*p* > 0.05, Table S2). Based on the MR-PRESSO global test, 11 MR associations were found to have pleiotropy (*p* < 0.05, Table S3); the outliers identified in each MR association are displayed in Table S4. Among which, the positive effect of childhood BMI (β: 0.497, 95% CI: 0.413, 0.581; *p* < 0.001) and the negative effect of menarche age (β: −0.083, 95% CI: −0.162, −0.003; *p* = 0.043) on the risk of T2D were reduced after removing these identified outliers; other obtained causal estimates were not distorted (*p* > 0.05, Table S3). Therefore, the MR results used in this study were not biased by pleiotropy. The causal direction found in this study was further detected and confirmed by the MR Steiger test (Table S3). Leave-one-out results showed that no individual IV exerted a substantial influence on the overall causal estimate (Figure S1). Moreover, our MR findings were generally consistent across multiple methods (Table S5). Taken together, these findings collectively suggest that the MR results are reliable and robust.

## 4. Discussion

By employing a two-sample MR design, this present study confirmed a causal effect of childhood obesity on early puberty onset in girls, while no such relationship was observed in boys. Additionally, a mediation analysis indicated that early puberty onset acts as a mediator in the association between childhood obesity and increased risk of T2D and CVD. Furthermore, this analysis highlighted the partial mediation effect of creatine on the relationship between childhood obesity and early puberty onset in girls.

Previous epidemiological studies and genetic evidence have consistently indicated a causal association between obesity in girls and earlier puberty onset [7,8,10,42,43,44]. Our study confirmed that childhood obesity and increased BMI contributed to a younger age of menarche and increased Tanner stage in girls, offering further support for these conclusions. Furthermore, we found that the increased Tanner stage in girls and a younger age of menarche play a mediating role in the causal pathway from childhood obesity to an increased risk of T2D. Additionally, a younger age of menarche partially mediated the positive effect of childhood obesity on the increased risk of CVD [17,18,19,20]. These findings align with previous research and suggest that maintaining a normal weight during childhood contributes to preventing early puberty and further reducing the future risk of T2D and CVD in adulthood. However, our study found no correlation between obesity and boys’ puberty onset, which was consistent with several studies [13,14]. This result prompts us to consider that the conflicting results from other observational evidence may be attributed to potential confounding effects or reverse causation [15,45], highlighting the necessity for further validation and investigation. 

Our MR results also demonstrated the causal effects of obesity on metabolic dysfunction and identified key obesity-related metabolites. The branched-chain amino acids (BCAAs) including valine, leucine, and isoleucine are essential amino acids characterized by their branched functional R groups. The concentration of BCAAs has been found to be increased in individuals with obesity, and was positively correlated with the visceral fat and insulin resistance [46]. The reduced expression of the BCKDH enzyme in obese individuals suggested that the elevated levels of BCAAs may be due to impaired metabolic processes [47]. Consistent with this, our MR results confirmed the positive effect of childhood obesity and higher BMI on valine and leucine levels, highlighting their crucial role in obesity. We also found that obesity plays a role in regulating the levels of gut-microbe-derived metabolites such as hippurate and trimethylamine. Hippurate is a typical constituent of urine that has been found to have a robust correlation with diet and the gut microbiota. Calvani et al. observed a significant decline in hippurate levels among obese individuals compared to healthy controls, and highlighted its potential as the most crucial discriminant metabolite [33]. Furthermore, a similar decrease was also observed in obese rats [48]. Our study confirmed the negative effect of obesity on hippurate levels as well. Trimethylamine is also a metabolite produced by gut bacteria during the metabolism of certain dietary compounds, which can be further converted into trimethylamine oxide in the liver. The evidence regarding the correlation between obesity and trimethylamine oxide is limited and remains controversial. Heianza et al. demonstrated a positive correlation between serum trimethylamine oxide levels and body fat distribution [49]. Furthermore, a meta-analysis found that a higher BMI was significantly correlated with an increase in serum trimethylamine oxide levels [50], whereas a systematic review reported no significant associations between serum trimethylamine oxide levels and various obesity-related indices, including body composition, BMI, and body fat distribution, after adjusting for all confounding factors [51]. Our MR analysis provided evidence of a causal link between childhood obesity and higher urinary levels of trimethylamine oxide. Additionally, childhood obesity was found to exert a negative effect on urine levels of trimethylamine, the precursor of trimethylamine oxide. Further research is needed to validate this relationship and to gain a comprehensive understanding of the underlying mechanisms. 

We also identified a causal link between childhood obesity/BMI and increased creatine levels. Creatine is a naturally occurring substance that is derived through both dietary intake and synthesized endogenously using arginine and glycine via the enzymes guanidinoacetate methyl transferase (GAMT) and arginine glycine amidinotransferase (AGAT) [52]. It is primarily stored within skeletal muscle cells and involved in energy metabolism processes. Creatine kinase enzymes catalyze the reversible transfer of high-energy phosphate groups between creatine and adenosine triphosphate (ATP), forming phosphocreatine and adenosine diphosphate (ADP). Therefore, the creatine kinase/phosphocreatine system is essential for maintaining cellular energy homeostasis and optimizing energy utilization in various physiological processes [53]. Although no previous study has reported creatine levels in obese individuals, creatine has been found to be involved in modulating the adipocyte thermogenic capacity and body weight of mammals [54]. A deficiency in phosphorylated creatine has been shown to protect against the development of obesity-induced metabolic syndrome [55]. Our study showed that obesity contributed the increased creatine levels, supporting these above conclusions. Furthermore, our mediation analysis provided evidence that increased creatine also plays a mediating role in the obesity-induced increase in the Tanner stage in girls.

Due to the significant energy demands associated with sexual maturation, metabolic factors are essential in conveying the energy status to the central regulatory center of reproductive function, hypothalamic kisspeptin neurons, which regulate the GnRH release and, ultimately, control the timing of puberty onset [34,35]. Among which, AMPK and mTOR are energy sensors that are highly expressed in the hypothalamus, and they play a crucial role in regulating the metabolic modulation of puberty. AMPK can be triggered in response to insufficient energy supply conditions, leading to delayed puberty onset. Accumulating evidence in rodent studies has demonstrated that chronic undernutrition- and adiponectin-induced AMPK stimulation delays puberty onset via the suppression of *Kiss1* gene expression and by further inhibiting GnRH release and GnRH-stimulated LH levels [34,56,57,58], while this delay can be largely prevented in Kiss1-specific AMPK knockout mice [34]. In contrast to AMPK, mTOR is activated in situations of energy excess that promote the onset and development of puberty. Activation of mTOR activates the HPG axis and advances the onset of puberty, reversing some of the permissive effects of leptin on pubertal onset and partially reversing the undernutrition-induced reduction in gonadotropin levels and puberty [35,59], while inhibiting mTOR activity delays puberty onset by reducing *Kiss1* gene expression in the arcuate nucleus and lowering the circulating levels of LH and estradiol in female rats [35,59]. It is noteworthy that creatine regulates both the AMPK and mTOR pathways. Reduced creatine biosynthesis induced by AGAT deficiency could lead to intracellular energy depletion and further stimulate AMPK activation in the skeletal muscles of mice [55]. Supplementation with creatine nitrate, a novel form of creatine, enhances the energy-buffering capacity of the creatine/phosphocreatine pool in the muscle, leading to a decreased AMP/ATP ratio, and further inhibits AMPK pathway activation by downregulating the upstream kinase (LKB1) [60]. Creatine supplementation can also enhance mTOR signaling by inducing the secretion of IGF-1, which binds to the IGF-1 receptor and initiates downstream activation of the phosphatidylinositol-3-kinase (PI3K)-AKT signaling pathway, ultimately resulting in the phosphorylation of mTOR [61,62,63,64]. Dysregulation of creatine transporter activity can induce an energy imbalance between the AMPK and mTOR pathways [53]. Hence, we hypothesized that increased creatine levels induced by childhood obesity could potentially promote early puberty onset of girls by inhibiting the AMPK signaling pathway and activating the mTOR signaling pathway and further stimulating the *kiss1 gene* expression, and GnRH and LH release, ultimately, increased the Tanner stage of girls (Figure 6B). In addition, glucose is inversely correlated with AMPK [65] and has also been causally associated with younger menarche age, further emphasizing the importance of energy homeostasis in the relationship between obesity and pubertal development.

Interestingly, a sex difference was found in creatine metabolism. The endogenous creatine store in females was lower than in males [52]. Estrogen and testosterone have been found to modulate creatine synthesis by regulating the expression of AGAT [66]. Additionally, estrogen and progesterone have the potential to modulate the activity of creatine kinase as well as the expression of essential enzymes involved in endogenous creatine biosynthesis [67]. Further research is needed to investigate whether this sex difference in creatine metabolism involves the sex-specific effect of obesity on the pubertal development.

## 5. Conclusions

Taken together, our study found a gender disparity in the association between childhood obesity and pubertal development. Childhood obesity did not have a causal effect on pubertal development in boys, but it did contribute to early puberty onset in girls and increased risk of T2D and CVD in adulthood. Moreover, creatine potentially served as a mediator in the causal pathway from obesity to early puberty onset in girls, while early puberty onset in girls played a mediating role in the pathway linking childhood obesity to increased risk of T2D and CVD. Our findings suggested that preventing childhood obesity holds potential advantages in averting early puberty onset in girls and reducing the risk of T2D and CVD in adulthood. Further investigation is needed to determine the involvement and specific role of creatine in this process.

## Figures and Tables

**Table 1:** Detailed information of GWAS summary statistics data.

Phenotype	nSNPs	Sample Size (Case)	Consortium	PMID
Obesity				
Childhood obesity	2,442,739	5530 (8318)	EGG	22484627
Childhood BMI	8,173,382	39,620	EGG	33045005
Tanner stage				
Tanner stage of boys	2,183,191	3769	EGG	24770850
Tanner stage of girls	2,183,191	6147	EGG	24770850
Age of menarche	9,851,867	243,944	MRC-IEU	NA
Childhood urinary metabolite	6,143,757	996	HELIX	33283231
T2D	24,167,560	490,089 (38,841)	GWAS catalog	34594039
CVD	11,973,400	459,324 (146,524)	UK Biobank	29892013

PMID, Pubmed ID; BMI, body mass index, nSNPs, the number of single-nucleotide polymorphisms; ECG, Early Growth Genetics Consortium; HELIX, Human Early Life Exposome; T2D, type 2 diabetes; CVD, cardiovascular disease.

## Data Availability

Data of childhood BMI, childhood obesity, and Tanner stage of girls and boys were obtained from the ECG (http://egg-consortium.org/, (accessed on 10 October 2023)). Data for age of menarche and urinary metabolites were sourced from the MRC-IE (http://www.ewascatalog.org/, (accessed on 10 October 2023)) and the HELIX (https://helixomics.isglobal.org/, (accessed on 10 October 2023)), respectively. Data for T2D and CVD were obtained from the GWAS Catalog (https://www.ebi.ac.uk/gwas/, (accessed on 15 February 2024)) with the accession IDs GCST90018926 and GCST90029019, respectively.

## Supplementary Materials

The following supporting information can be downloaded at: https://www.mdpi.com/article/10.3390/metabo14030137/s1, Figure S1: Leave-one-out analysis confirmed the robustness of Mendelian randomization results; Table S1: The mediation analysis results; Table S2: Instrumental variables and harmonized data used in Mendelian randomization analysis; Table S3: Sensitive analysis in Mendelian randomization; Table S4: Outliers identified by MR-PRESSO in Mendelian randomization analysis; Table S5: Full result of Mendelian randomization estimates using multiple methods.
