# Supplementary figures and images for: Creatine Acts as a Mediator of the Causal Effect of Obesity on Puberty Onset in Girls: Evidence from Mediation Mendelian Randomization Study
Source: Metabolites. 2024 Feb 25;14(3):137. doi: 10.3390/metabo14030137 (PMC10971908; doi:10.3390/metabo14030137)

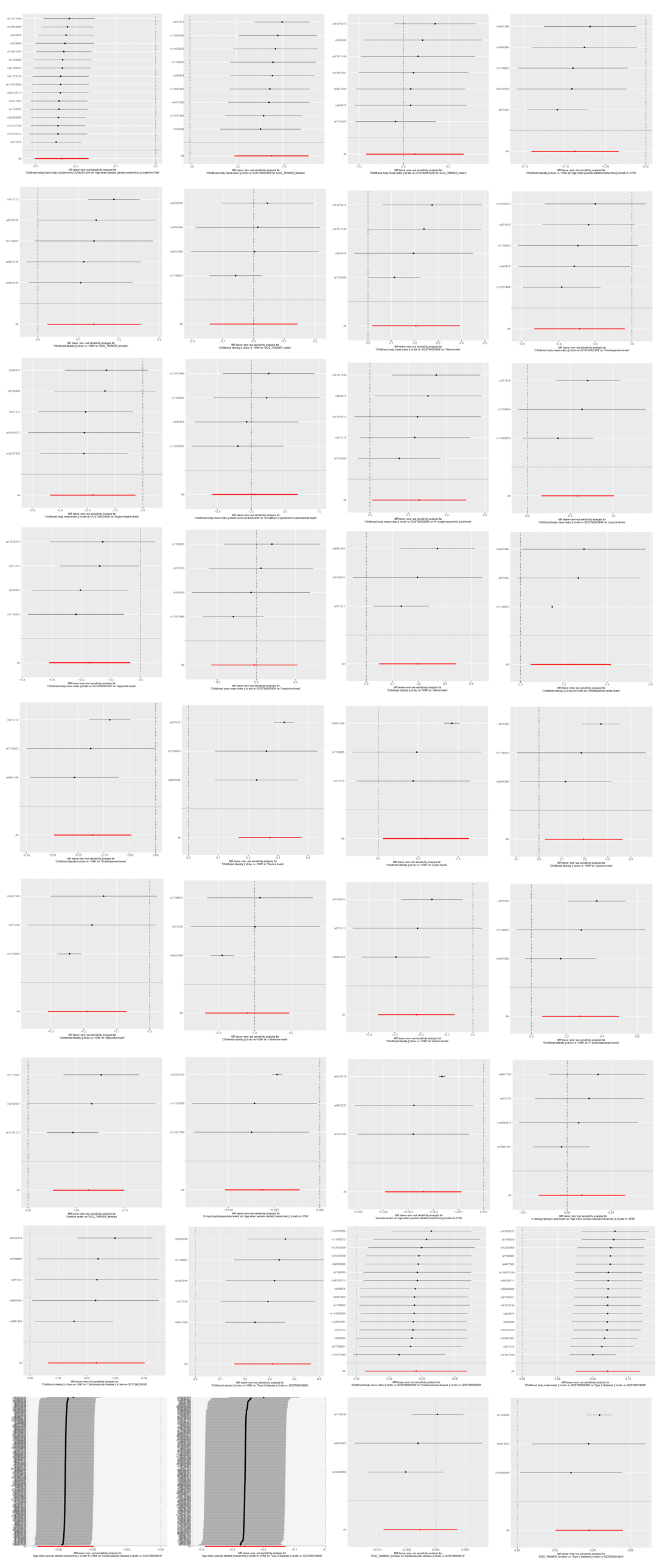

Supplement: Supplementary file 1 [file metabolites-14-00137-s001.zip › Figure S1.pdf]
